# Understanding side-effects of anti-CGRP and anti-CGRP receptor antibodies

**DOI:** 10.1186/s10194-020-01097-3

**Published:** 2020-03-16

**Authors:** Kristian Agmund Haanes, Lars Edvinsson, Anette Sams

**Affiliations:** 1 Department of Clinical Experimental Research, Copenhagen University Hospital, Glostrup, Denmark; 2 Department of Clinical Sciences, Division of Experimental Vascular Research, Lund University, Lund, Sweden

**Keywords:** CGRP, GLP-1, Side effects, Constipation, Monoclonal antibodies

To the editor.

Therapeutic prevention and intervention of migraine attacks with the novel monoclonal antibodies (mAbs) against Calcitonin Gene-Related Peptide (CGRP) or the CGRP-receptor (hereafter named jointly “anti-CGRP therapy”) are currently being initiated in thousands of patients.

Although the clinical trials on anti-CGRP therapy have reported surprising limited number of side-effects considering CGRPs abundance [1], the most up to date (31th of December 2019) data from the FDA Adverse Event Reporting System, show that 4082 of the total reported side-effects (24,573) to date were related to gastrointestinal disorders (17%). Generally, constipation is a significant anti-CGRP side-effect reported in 3–4% of patients [2]. These effects might be exacerbated when patients are using other medications that also affect the gastrointestinal system, such as anti-depressants and morphine receptor activating drugs.

Reviewing the current literature on the importance of CGRP in the gastrointestinal system, it appears that CGRP per se may reduce gastric emptying [3, 4], abrogate contractions in rat colon [5] and reduce food intake [6, 7]. Some of these effects were indirect via neuronal CGRP-receptors [3] and others were local [5]. This letter aims to raise the awareness of an important gastrointestinal target for CGRP which could explain anti-CGRP therapy induced constipation and potentially suggest a pharmacological intervention to relieve symptoms for patients that are suffering from constipation.

Sams and coworkers have shown that a long-acting CGRP peptide analogue significantly increased levels of circulating Glucagon-Like Peptide-1 (GLP-1) by > 60% [6]. Associated studies in a murine enteroendocrine L-cell line [6] and on perfused ileum [8] supported the direct and specific concentration-dependent CGRP-induced GLP-1 secretion [6]. Thus, since CGRP induces local GLP-1 secretion in the gastrointestinal tract, reduced levels of GLP-1 could be associated with anti-CGRP therapy. The regulation might be bidirectional since GLP-1 has been shown to induce secretion of enteric nerve CGRP in mouse colon and via modulation of epithelial salt conductance, this has been suggested as a mechanism of GLP-1 drug induced diarrhea [9].

Since the above studies collectively show that gastrointestinal side-effects are most likely indirect, they could therefore be treated without interfering with the clinical effect of blocking the CGRP signaling system. In addition, these data provide important insight to the effects of CGRP in the gastrointestinal system, and we recommend that GLP-1 and GLP-1 metabolite levels should be investigated clinically in patients on the anti-CGRP or CGRP-receptor mAbs. Such investigation is highly relevant in the light of the importance of GLP-1 in long term glucose homeostasis, regulation of food-intake and gastric motility [10] and could provide further insight to conclude whether GLP-1 supplementation would be beneficial in a subgroup of patients suffering from migraine-therapy associated constipation.

## Data Availability

Not applicable.

## Abbreviations

CGRP: Calcitonin Gene-Related Peptide
mAbs: Monoclonal antibodies
GLP-1: Glucagon-Like Peptide-1
